# Coordinated targeting of cold and nicotinic receptors synergistically improves obesity and type 2 diabetes

**DOI:** 10.1038/s41467-018-06769-y

**Published:** 2018-10-23

**Authors:** Christoffer Clemmensen, Sigrid Jall, Maximilian Kleinert, Carmelo Quarta, Tim Gruber, Josefine Reber, Stephan Sachs, Katrin Fischer, Annette Feuchtinger, Angelos Karlas, Stephanie E. Simonds, Gerald Grandl, Daniela Loher, Eva Sanchez-Quant, Susanne Keipert, Martin Jastroch, Susanna M. Hofmann, Emmani B. M. Nascimento, Patrick Schrauwen, Vasilis Ntziachristos, Michael A. Cowley, Brian Finan, Timo D. Müller, Matthias H. Tschöp

**Affiliations:** 1 Institute for Diabetes and Obesity, Helmholtz Diabetes Center (HDC), Helmholtz Zentrum Muenchen & German Center for Diabetes Research (DZD), Neuherberg, Germany; 2 Novo Nordisk Foundation Center for Basic Metabolic Research, Faculty of Health and Medical Sciences, University of Copenhagen, Copenhagen, Denmark; 3 Division of Metabolic Diseases, Department of Medicine, Technische Universität München, Munich, Germany; 4 Section for Molecular Physiology, Department of Nutrition, Exercise and Sports, Faculty of Science, University of Copenhagen, Copenhagen, Denmark; 5 Institute of Biological and Medical Imaging, Helmholtz Zentrum München, German Research Center for Environmental Health (GmbH), Neuherberg, Germany; 6 Chair for Biological Imaging, Technical University of Munich, Munich, Germany; 7 Institute of Diabetes and Regeneration Research, Helmholtz Zentrum Muenchen, German Research Center for Environmental Health (GmbH), Neuherberg, Germany; 8 German Center for Diabetes Research (DZD), Neuherberg, Germany; 9 Medizinische Klinik und Poliklinik IV, Klinikum der Ludwig Maximilian Universität (LMU), Munich, Germany; 10 Research Unit Analytical Pathology, Helmholtz Zentrum München, Neuherberg, Germany; 11 Department of Physiology, and Biomedicine Discovery Institute, Monash University, Clayton, Australia; 12 Department of Human Biology and Human Movement Sciences, NUTRIM School for Nutrition and Translational Research in Metabolism, Maastricht University Medical Center, Maastricht, Netherlands

## Abstract

Pharmacological stimulation of brown adipose tissue (BAT) thermogenesis to increase energy expenditure is progressively being pursued as a viable anti-obesity strategy. Here, we report that pharmacological activation of the cold receptor transient receptor potential cation channel subfamily M member 8 (TRPM8) with agonist icilin mimics the metabolic benefits of cold exposure. In diet-induced obese (DIO) mice, treatment with icilin enhances energy expenditure, and decreases body weight, without affecting food intake. To further potentiate the thermogenic action profile of icilin and add complementary anorexigenic mechanisms, we set out to identify pharmacological partners next to icilin. To that end, we specifically targeted nicotinic acetylcholine receptor (nAChR) subtype alpha3beta4 (α3β4), which we had recognized as a potential regulator of energy homeostasis and glucose metabolism. Combinatorial targeting of TRPM8 and nAChR α3β4 by icilin and dimethylphenylpiperazinium (DMPP) orchestrates synergistic anorexic and thermogenic pathways to reverse diet-induced obesity, dyslipidemia, and glucose intolerance in DIO mice.

## Abstract (web summary)

Tobacco smoking and cold exposure are environmental modulators of human energy metabolism suppressing appetite and increasing energy expenditure, respectively. Here, the authors develop a novel pharmacological strategy in which they simultaneously mimic the metabolic benefits of both phenomena through small-molecule combination therapy, and show that this treatment improves metabolic health of obese mice.

## Introduction

Successful management of the global obesity epidemic is required to halt the increasing prevalence of cardiometabolic diseases^1^. Pharmacological stimulation of metabolic rate was explored as a weight-lowering strategy in the first half of the 20th century. However, harmful side effects, such as excessive heat production and microvascular disease, following chronic administration of drugs such as 2,4-dinitrophenol (DNP) may have contributed to direct the anti-obesity field towards appetite-suppressants^2^. The now decade-old observation that humans possess recruitable brown adipose tissue (BAT) that generates heat in response to cold stress^3–5^ reinvigorated substantial research on BAT thermogenesis and anti-obesity therapeutic strategies aimed to increase energy expenditure.

A family of thermoreceptors, the transient receptor potential (TRP) channels, are expressed in afferent dorsal root ganglion (DRG) sensory neurons, and primarily function to convey information of environmental temperature to the central nervous system (CNS)^6^. The transient receptor potential cation channel subfamily M member 8 (TRPM8) is a proximate regulator of the cold-sensing cascade that culminates in the induction of BAT thermogenesis to defend body temperature in response to environmental cold^7^. TRPM8 can also be activated by chemical ligands such as menthol and the high-potency small molecule icilin^7,8^. Menthol-induced TRPM8 activation increases body temperature^9^, BAT uncoupling protein 1 (UCP1) expression, and protects mice from diet-induced obesity^10^, underlining the potential of developing TRPM8-based pharmacotherapies to treat obesity.

Because interventions that increase energy expenditure, including cold exposure, frequently elicit counter-regulatory induction of orexigenic feeding circuits^11,12^, complementary anorexigenic actions are likely required to chronically reverse metabolic imbalances. Here, we hypothesized that the nicotinic acetylcholine receptor (nAChR) subtype α3β4 would be an ideal pharmacological partner to TRPM8 agonism by capitalizing on the well-described effects of smoking to suppress hunger. Pharmacological stimulation of hypothalamic α3β4 nAChRs suppresses appetite via the hypothalamic-melanocortin system^13^. Additionally, the nAChR α3β4 is the primary ganglionic nAChR that upon pharmacological stimulation depolarizes BAT and increases lipolysis^14–16^, and somewhat paradoxically, nAChR activation also acts centrally to lower body temperature^17,18^. Therefore, we hypothesized that pharmacological stimulation of α3β4 will complement the thermogenic virtues of TRPM8 agonism while appropriately counter-balancing systemic hyperthermia and potential counter-regulatory hyperphagia. Thus, we demonstrate that dual nAChR α3β4 and TRPM8 agonism, which in essence biochemically mimics the two environmental stimuli known to improve systemic metabolism—cold exposure and cigarette smoking, evokes a cascade of physiological nodes that harmonize to reverse obesity and type 2 diabetes

We report that a potent TRPM8 agonist, icilin, enhances energy expenditure to lower body weight in diet-induced obese (DIO) mice. We discovered that combining icilin with a α3β4 nAChR agonist dimethylphenylpiperazinium (DMPP) elicits coordinated metabolic actions that synergize to lower body weight, correct glucose intolerance, and reduce hepatic steatosis. Mechanistically, we reveal that central melanocortin signaling as well as sympathetic nervous system-linked thermogenesis are indispensable for the orchestration of the complete metabolic benefits of this novel combination strategy.

## Result

### Icilin increases energy expenditure and lowers body weight

**Fig. 1:**
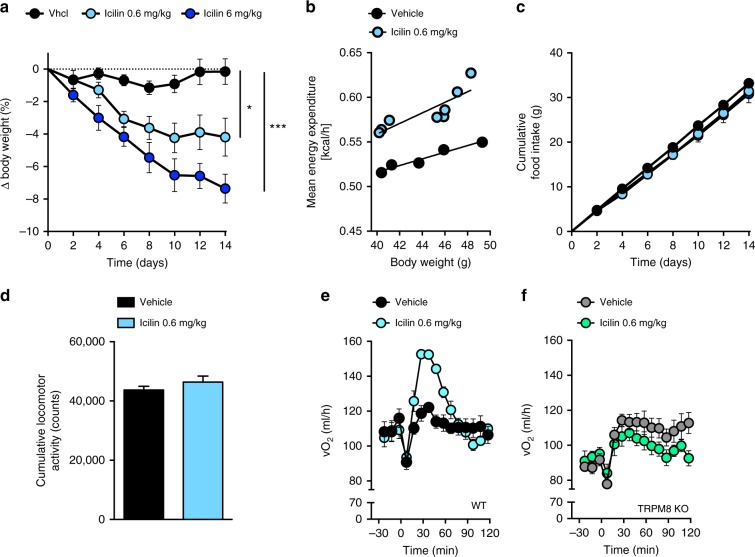
TRPM8 agonist icilin increases energy expenditure and lowers body weight in DIO mice. **a** Effects on body weight, **b** energy expenditure, **c** food intake, **d** locomotor activity following daily s.c. injections of vehicle (black, *n* = 8 for **a**, **c**; *n* = 5 for **b**, **d**), icilin 0.6 mg/kg (light blue, *n* = 8), and icilin 6 mg/kg (dark blue, *n* = 6) in DIO male C57Bl6j mice for 14 days. **e**, **f** Oxygen consumption following a single injection of vehicle (black/gray, *n* = 8) or icilin (blue/green, *n* = 8) to DIO male WT mice or DIO TRPM8 KO mice. *p < 0.05, ***p < 0.001 by two-way ANOVA (**a**, **c**) with Tukey post-hoc test and two-tailed Student’s *t*-test (**d**). All data are presented as mean ± SEM

To test if the TRPM8 agonist icilin mimics the metabolic benefits of cold exposure, we administered icilin to mice maintained on a high-fat, high sucrose diet (HFD). Chronic treatment with icilin for 14 days dose-dependently lowered body weight (Fig. 1a), reflected in a reduction in body fat (Supplementary Fig. 1a). This effect on body weight and fat mass was due to an increase in energy expenditure (Fig. 1b), while food intake (Fig. 1c) and locomotor activity were unaffected by icilin (Fig. 1d). In TRPM8 null mice, icilin had no effect on oxygen consumption rate (Fig. 1e, f) or weight loss (Supplementary Fig. 3d, f), underscoring that the ability of icilin to lower body weight by increasing energy expenditure requires functional TRPM8. In contrast to previous work^10,19^, we did not detect meaningful expression of *Trpm8* in BAT of mice housed at 30 °C or at 5 °C suggesting that the icilin effect on BAT energy expenditure is not explained by direct effects of icilin on adipocytes (Supplementary Fig. 1b). In agreement, icilin did not increase *Ucp1* transcription or oxygen consumption in mouse (Supplementary Fig. 1c–d) or human (Supplementary Fig. 1e–f) primary brown adipocytes, suggesting indirect actions to increase thermogenesis, likely through induction of sympathetic tone.

### DMPP improves diet-induced obesity and glucose intolerance

**Fig. 2:**
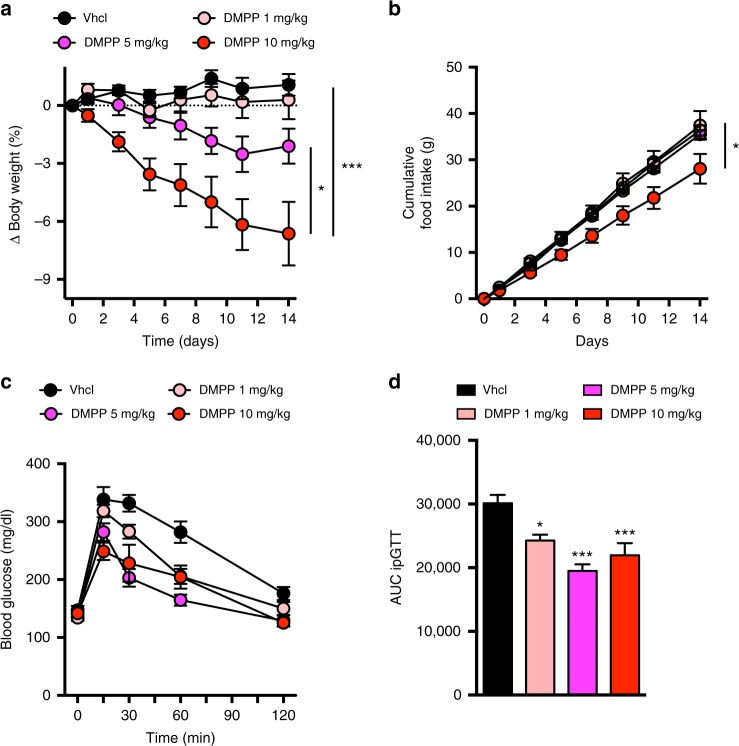
The nicotinic receptor agonist DMPP lowers body weight and improves glucose tolerance in DIO mice. **a** Effects on body weight, **b** food intake, and **c**, **d** glucose tolerance following daily s.c. injections to DIO male C57Bl6j mice of vehicle (black, *n* = 8 for **a**, **b**; *n* = 7 for **c**, **d**), DMPP 1 mg/kg (light red, *n* = 8), DMPP 5 mg/kg (pink, *n* = 7 for **a**, **b**; *n* = 8 for **c**, **d**), and DMPP 10 mg/kg (dark red, *n* = 7) for 14 days. *p < 0.05, ***p < 0.001 by two-way ANOVA (**a**, **b**, **c**) and one-way ANOVA with Tukey post-hoc test (**d**). All data are presented as mean ± SEM

Although the ability of icilin to increase energy expenditure seems to be appropriately uncoupled from compensatory hyperphagia, we hypothesized that additional pharmacology concurrently targeting central satiety circuits would complement TRPM8-based pharmacology to achieve greater weight loss. We discovered that the nicotinic receptor α3β4 agonist DMPP dose-dependently lowered body weight in DIO mice (Fig. 2a). Similar to nicotine (Supplementary Fig. 2a, b), DMPP reduced body weight by suppressing food intake (Fig. 2b). However, in contrast to nicotine (Supplementary Fig. 2c, d), DMPP markedly improved diet-induced glucose intolerance, even at doses with negligible effect on body weight change (Fig. 2c, d).

### Icilin and DMPP synergistically lower body weight

**Fig. 3:**
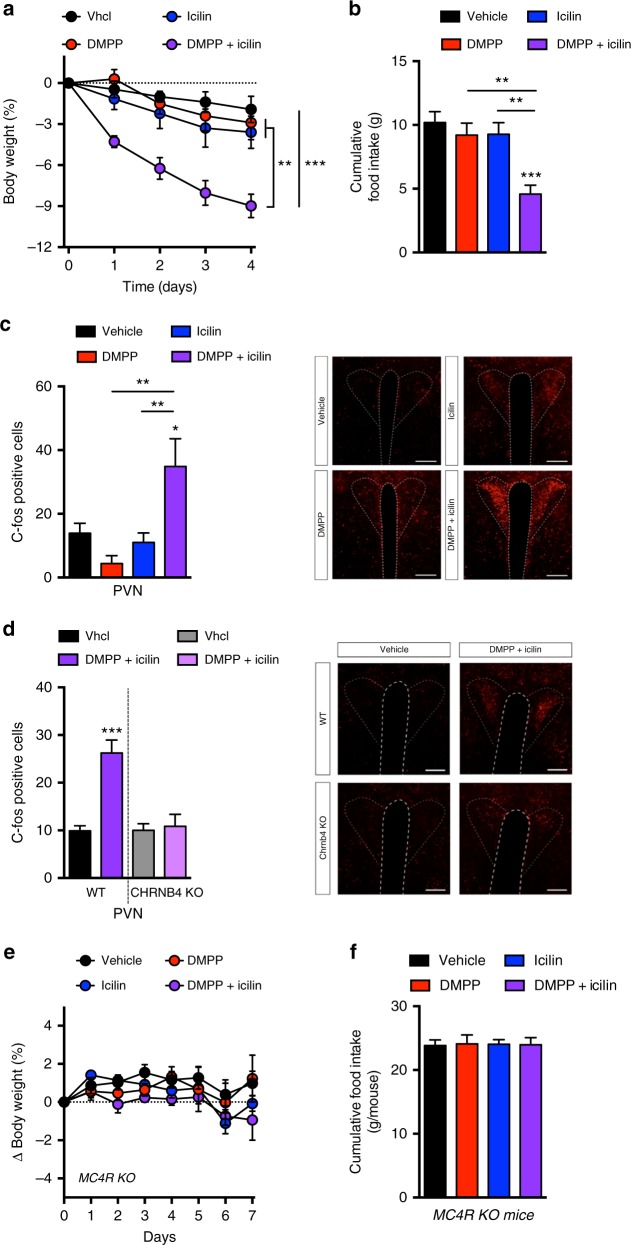
Icilin and DMPP synergistically lower body weight in DIO mice via central mechanisms. **a** Effects on body weight, **b** cumulative food intake, and **c** cFOS positive neurons in the hypothalamus following daily s.c. injections to DIO male C57Bl6j mice of vehicle (black, n = 8 for **a**, **b**; *n* = 6 for **c**), DMPP 10 mg/kg (red, *n* = 8 for **a**, **b**; *n* = 6 for **c**), icilin 5 mg/kg (blue, *n* = 8 for **a**, **b**; *n* = 6 for **c**), or the combination of DMPP 10 mg/kg and icilin 5 mg/kg (purple, *n* = 7 for **a**, **b**; *n* = 8 for **c**) for 4 days (**a**, **b**) or 3 days (**c**), respectively. **d** cFOS positive neurons in the hypothalamus following daily s.c. injections to DIO male WT C57Bl6j mice or to DIO male CHRNB4 KO mice of vehicle (black, *n* = 5/gray, *n* = 4) or the combination of DMPP 10 mg/kg and icilin 5 mg/kg (purple, *n* = 5/light purple, *n* = 5) for 3 days. **e** Effects on body weight and **f** cumulative food intake following daily s.c. injections to HFD-fed male MC4R KO mice of vehicle (black, *n* = 8), DMPP 5 mg/kg (red, *n* = 8), icilin 5 mg/kg (blue, *n* = 8), or the combination of DMPP 5 mg/kg and icilin 5 mg/kg (purple, *n* = 7) for 7 days. All scale bars are 100 µm. *p < 0.05, **p < 0.01, ***p < 0.001 by two-way ANOVA (**a**, **e**) and one-way ANOVA with Tukey post-hoc test (**b**, **c**, **d**, **f**). All data are presented as mean ± SEM

Following the results with icilin and DMPP monotherapy on energy expenditure and appetite, respectively, we tested icilin and DMPP in combination as a single formulation. Cotreatment with icilin (5 mg/kg) and DMPP (10 mg/kg) synergistically lowered body weight in DIO mice (Fig. 3a) coinciding with a robust reduction in food intake (Fig. 3b) and increased energy expenditure (Supplementary Fig. 3a). The synergistic weight-lowering effects of DMPP and icilin cotreatment were corroborated by chronic treatment studies (Fig. 5a, Supplementary Fig. 3b). To explore the central hypothalamic actions of the combination therapy, we assessed c-FOS immune reactivity as an indirect measure of neuronal activity. c-FOS staining in the paraventricular nucleus (PVN) was increased in mice treated with the combination of icilin and DMPP, but not in response to either monotherapy (Fig. 3c). The induction of c-FOS in the PVN following the combination therapy was absent in nAChR β4 KO mice (Fig. 3d), suggesting that α3β4 nAChRs are necessary for the anorexigenic effects of the treatment. In support, DIO nAChR β4 KO mice did not lose body weight in response to DMPP and icilin cotreatment (Supplementary Fig. 3h).

The notion that the satiety benefits of the combination treatment specifically implicate the PVN prompted us to investigate the role of the leptin–melanocortin system to the anorexigenic phenotype. We observed a complete loss-of-effect in DIO melanocortin-4 receptor (MC4R) KO mice following treatment with both the monotherapies and the combination of icilin and DMPP (Fig. 3e, f), underlining that the melanocortin pathway is indispensable for the weight-lowering benefits of the cotreatment.

### Icilin and DMPP stimulate thermogenic pathways

**Fig. 4:**
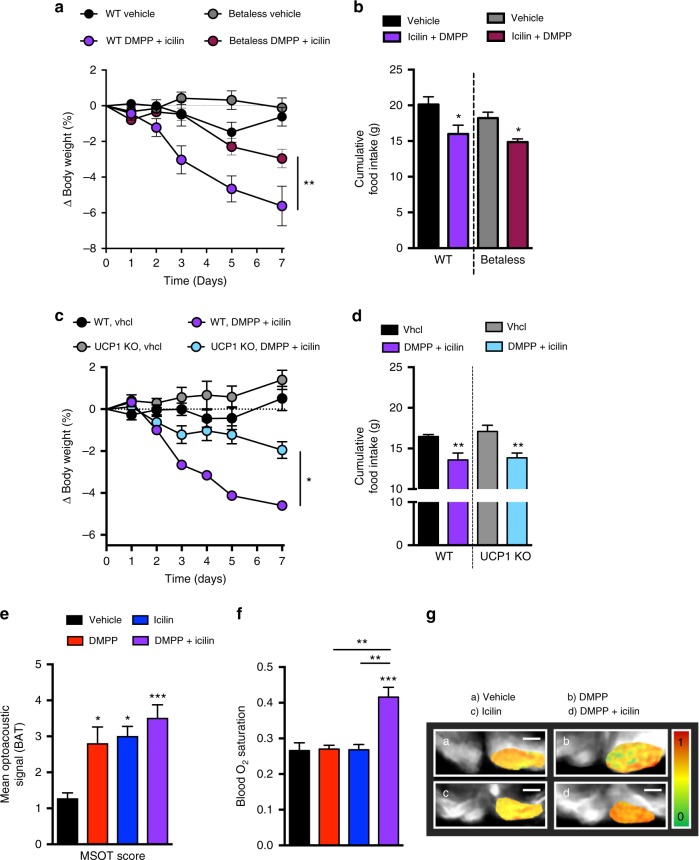
Icilin and DMPP combination therapy increases sympathetic nervous system-linked BAT thermogenesis. **a** Effects on body weight and **b** cumulative food intake following daily s.c. injections to DIO male betaless mice or DIO male WT C57Bl6j mice of vehicle (black, *n* = 8/gray, *n* = 8) or the combination of DMPP 5 mg/kg and icilin 5 mg/kg (purple, *n* = 8/bordeaux, *n* = 7) for 7 days. **c** Effects on body weight and **d** cumulative food intake following daily s.c. injections to global UCP1 KO DIO male mice or DIO male C57Bl6j control mice of vehicle (black, *n* = 7/gray, *n* = 7) or the combination of DMPP 5 mg/kg and icilin 5 mg/kg (purple, *n* = 7/light blue, *n* = 7) for 7 days. **e** Mean optoacoustic signal intensity and **f** blood oxygen saturation (SO_2_) measured by Multi-Spectral Optoacoustic Tomography (MSOT) in selected BAT regions of male C57Bl6j control mice of vehicle (black, *n* = 6), DMPP 10 mg/kg (red, *n* = 6), icilin 5 mg/kg (blue, *n* = 6), or the combination of DMPP 10 mg/kg and icilin 5 mg/kg (purple, *n* = 6). (**g**–**a** to **g**–**d**) MSOT image of BAT at 800 nm (gray scale) with overlaid of SO_2_ (green-red scale) in: a control- (**g**–**a**), DMPP- (**g**–**b**), icilin- (**g**–**c**), and combination-injected mouse (g-d). All scale bars are 5 mm.*p < 0.05, **p < 0.01, ***p < 0.001 by two-way ANOVA (**a**, **c**), one-way ANOVA (**e**, **f**) with Tukey post-hoc test and two-tailed Student’s *t*-test (within genotype) (**b**, **d**). All data are presented as mean ± SEM

To gain insight into the mechanisms responsible for the energy expenditure induction downstream of the MC4R, we tested the ability of DMPP and icilin to reverse obesity in mice lacking β-adrenergic receptors (betaless mice). The weight loss following icilin and DMPP treatment was significantly blunted in the betaless mice (Fig. 4a), while food intake was suppressed (Fig. 4b), indicating the contribution of the adrenergic system to selectively mediate the effects on energy expenditure. Except for a subtle increase in brown fat *Pgc1a* mRNA in response to icilin and the cotreatment (Supplementary Fig. 5a), brown fat and inguinal white adipose tissue gene programs of thermogenesis, mitochondrial transport, creatine metabolism, and proteasomal activity were unaffected in treated betaless mice (Supplementary Fig. 5a–h). To assess the role of UCP1-mediated thermogenesis, we tested the combination of icilin and DMPP in UCP1 KO and WT mice. The body weight-lowering effect was blunted in UCP1 KO mice (Fig. 4c), while as observed in the betaless mice, food intake was still suppressed (Fig. 4d). We also confirmed the necessity of functional TRPM8 for DMPP and icilin-mediated reductions in body fat and weight (Supplementary Fig. 3b–g). Using Multi-Spectral Optoacoustic Tomography (MSOT) imaging of the BAT in vivo^20^, we detected an increase in the optoacoustic signal intensity over the BAT region following both icilin and DMPP monotherapy. Coadministration of both molecules resulted in the largest increase (Fig. 4e). Only the combination of icilin and DMPP increased oxygen saturation of the delivered blood to the BAT (Fig. 4f–g), emphasizing that the combination of icilin and DMPP is superior to the monotherapies in delivering oxygen for metabolic processes in the brown fat. Collectively, these data underscore that thermogenic pathways utilizing SNS-driven activation of β-ARs and UCP1 contribute substantially to the weight-lowering benefits of DMPP and icilin pharmacology.

### Synergistic pharmacology is independent of temperature

**Fig. 5:**
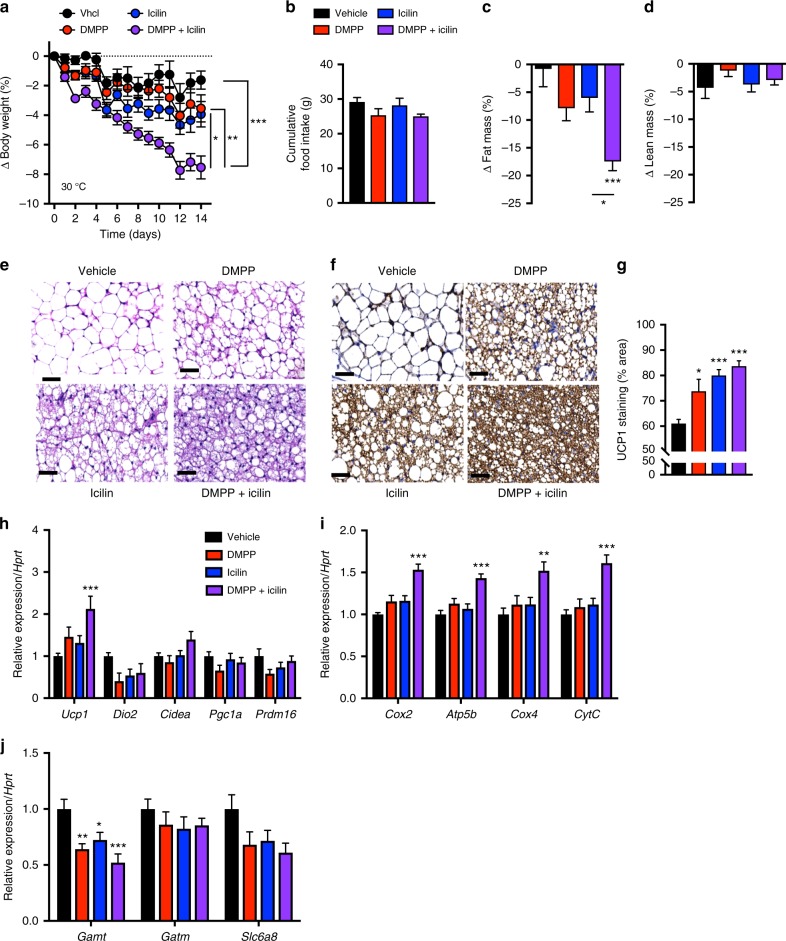
Icilin and DMPP reverse obesity and increase BAT UCP1 at thermoneutral conditions. **a** Effects on body weight, **b** cumulative food intake, changes in **c** fat mass and **d** lean mass, **e** BAT H & E staining, and **f** UCP1 immunoreactivity staining in BAT with **g** quantification of UCP1 immunoreactivity staining (% area). BAT expression of genes involved in **h** thermogenesis, **i** mitochondrial transport, and **j** creatine signaling pathway following daily s.c. injections of vehicle (black or white, *n* = 8), DMPP 5 mg/kg (red, *n* = 7), icilin 5 mg/kg (blue, *n* = 8), or the combination of DMPP 5 mg/kg and icilin 5 mg/kg (purple, *n* = 7) to DIO male C57Bl6j chronically housed at 30 °C for 14 days. All scale bars are 50 µm. *p < 0.05, **p < 0.01, ***p < 0.001 by two-way ANOVA (**a**) and one-way ANOVA (**b**, **c**, **d**, **g**, **h**, **i**, **j**) with Tukey post-hoc test. All data are presented as mean ± SEM

Because mice housed at thermoneutral conditions may more accurately model drug-induced thermogenesis in humans^21,22^, we tested DMPP and icilin cotreatment in DIO mice housed at 30 °C. Analogous to the effect at room temperature, we found that treatment of mice acclimated at 30 °C with the combination of DMPP and icilin synergistically lowered their body weight and fat mass in comparison with the monotherapies (Fig. 5a, c, d). Unlike the effect at room temperature, the weight loss of the mice housed at thermoneutrality was independent of a substantial reduction in food intake (Fig. 5b). DMPP and icilin monotherapies increased BAT multilocularity (Fig. 5e) and UCP1 protein (Fig. 5f, g), but not thermogenic gene programs in BAT (Fig. 5h) or inguinal white adipose tissue (iWAT) (Supplementary Fig. 6d). The combination of DMPP and icilin was superior in increasing BAT multilocularity (Fig. 5e), UCP1 protein levels (Fig. 5f, g), *Ucp1* mRNA, and genes involved in mitochondrial transport (Fig. 5h, i). DMPP monotherapy and its combination with icilin suppressed the expression of *Gamt*, an enzyme involved in creatine synthesis (Fig. 5j). No effects were observed on iWAT beiging (Supplementary Fig. 6b–c) or thermogenic gene programs in the iWAT of treated mice (Supplementary Fig. 6d–g). These findings underscore that the thermogenic complementary effects of dual TRPM8 and nAChR activation are preserved in DIO mice housed at thermoneutral conditions.

### Icilin and DMPP improve glucose control and fatty liver disease

**Fig. 6:**
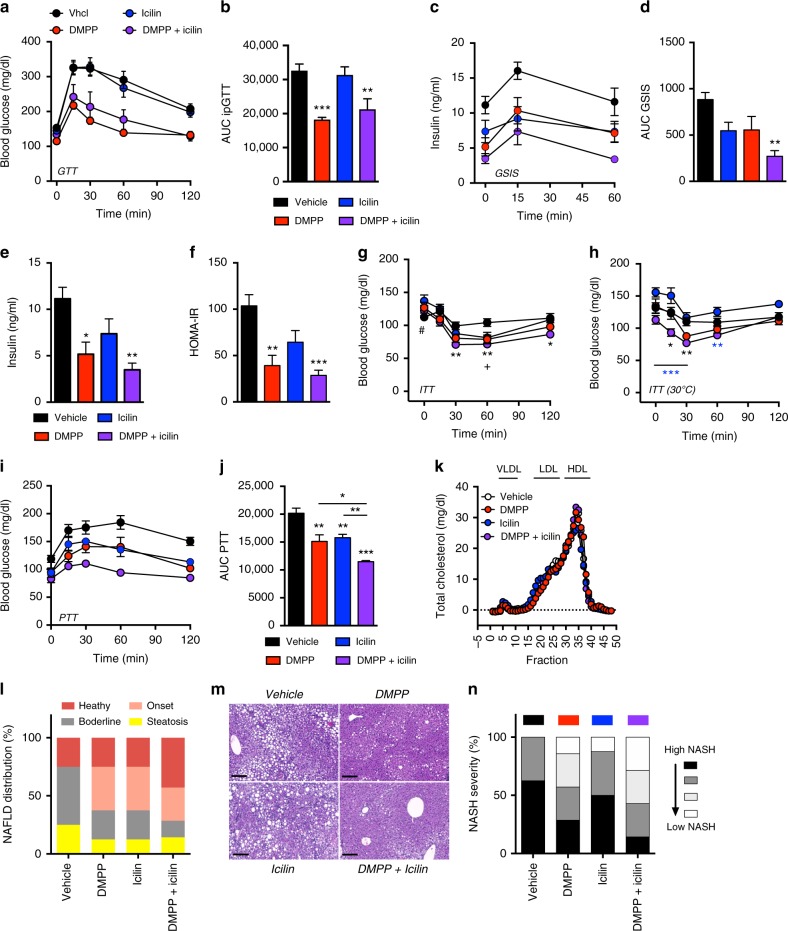
Icilin and DMPP reverse diet-induced glucose intolerance, insulin resistance, and hepatic steatosis in DIO mice. **a**, **b** Effects on glucose tolerance and **c**, **d** glucose-stimulated insulin secretion following daily s.c. injections to DIO mice of vehicle (black, *n* = 8), DMPP 10 mg/kg (red, *n* = 8), icilin 5 mg/kg (blue, *n* = 8), or DMPP 10 mg/kg and icilin 5 mg/kg (purple, *n* = 7) for 7 days. **e** Effects on 6-h fasted insulin levels, **f** HOMA-IR score, and **g** insulin tolerance following daily s.c. injections to DIO mice of vehicle (black, *n* = 8), DMPP 10 mg/kg (red, *n* = 8), icilin 5 mg/kg (blue, *n* = 8), or DMPP 10 mg/kg and icilin 5 mg/kg (purple, *n* = 7) for 7 days at 23 °C. **h** Effect on insulin tolerance following 14 days of daily s.c. injections of vehicle (black, *n* = 8), DMPP 5 mg/kg (red, *n* = 7), icilin 5 mg/kg (blue, *n* = 8), or the combination of DMPP 5 mg/kg and icilin 5 mg/kg (purple, *n* = 7) in DIO mice housed at 30 °C. **i** and **j** Effect on pyruvate tolerance following daily s.c. injections to DIO mice of vehicle (black, *n* = 7), DMPP 5 mg/kg (red, *n* = 7), icilin 5 mg/kg (blue, *n* = 7), or DMPP 5 mg/kg and icilin 5 mg/kg (purple, *n* = 7) for 20 days. **k** Plasma lipoprotein fractions following daily s.c. injections to DIO mice of vehicle (black, *n* = 7), DMPP 5 mg/kg (red, *n* = 7), icilin 5 mg/kg (blue, *n* = 7), or DMPP 5 mg/kg and icilin 5 mg/kg (purple, *n* = 7) for 21 days. **l** Effect on hepatic steatosis score following daily s.c. injections to DIO mice housed at 23 °C of vehicle (*n* = 8), DMPP 10 mg/kg (*n* = 8), icilin 5 mg/kg (*n* = 8), or DMPP 10 mg/kg and icilin 5 mg/kg (*n* = 7) for 14 days. **m** Liver H & E stainings and **n** NASH severity (%) following daily s.c. injections to DIO mice housed at 30 °C of vehicle (black, *n* = 8), DMPP 5 mg/kg (red, *n* = 7), icilin 5 mg/kg (blue, *n* = 8), or DMPP 5 mg/kg and icilin 5 mg/kg (purple, *n* = 7) for 14 days. All scale bars are 200 µm. *p < 0.05, **p < 0.01, ***p < 0.001 by two-way ANOVA (**a**, **c**, **g**, **h**, **i**) and one-way ANOVA (**b**, **d**, **e**, **f**, **j**) with Tukey post-hoc test. ^#^*p* < 0.05 vehicle vs. icilin and ^+^*p* < 0.05 vehicle vs. DMPP (g). All data are presented as mean ± SEM

**Fig. 7:**
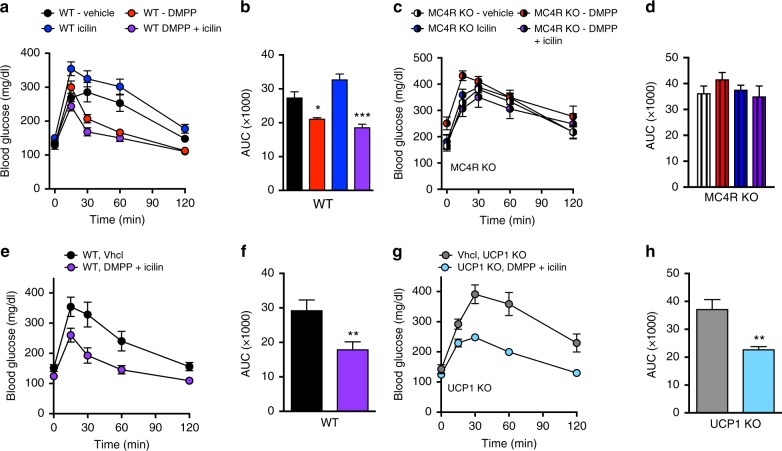
The melanocortin system is indispensable for the glycemic benefits of icilin and DMPP combination therapy. **a** and **b** Effects on glucose tolerance following daily s.c. injections to DIO male C57Bl6j mice or **c**, **d** MC4R KO mice of vehicle (black, *n* = 8/striped white, *n* = 8), DMPP 5 mg/kg (red, *n* = 8/striped red, *n* = 8), icilin 5 mg/kg (blue, n = 8/striped blue, *n* = 8), or the combination of DMPP 5 mg/kg and icilin 5 mg/kg (purple, *n* = 8/striped purple, *n* = 7) for 7 days. **e** and **f** Effects on glucose tolerance following daily s.c. injections to DIO male C57Bl6j mice or **g**, **h** UCP1 KO mice of vehicle (black, *n* = 7/gray, *n* = 7) or the combination of DMPP 5 mg/kg and icilin 5 mg/kg (purple, *n* = 7/light blue, *n* = 7) for 7 days. *p < 0.05, **p < 0.01, ***p < 0.001 by two-way ANOVA (**a**, **c**, **e**, **g**), one-way ANOVA wit Tukey post-hoc test (**b**, **d**), or two-tailed Student’s *t*-test (**f**, **h**). All data are presented as mean ± SEM

Whereas icilin monotherapy has negligible effects on glucose metabolism (Fig. 6a, b, Fig. 7a, Supplementary Fig. 8c), DMPP alone and in combination with icilin corrected diet-induced glucose intolerance following 1-weak of treatment (Fig. 6a, b). The insulin response during the glucose tolerance test (GTT) was lower following combination treatment relative to the monotherapies and vehicle control (Fig. 6c, d), implying enhanced insulin sensitivity. This notion was corroborated by HOMA-IR assessment (Fig. 6f) and by insulin tolerance tests (ITT) executed at both ambient room temperature (Fig. 6g) and at thermoneutral conditions (Fig. 6h) demonstrating superior effects of DMPP and icilin cotreatment relative to the monotherapies on insulin sensitivity. Notably, we observed opposing effects of icilin monotherapy on insulin sensitivity depending on the housing temperature. Hepatic glucose output was indirectly assessed by a pyruvate tolerance test (PTT). The glucose excursion during the PTT was substantially dampened by both icilin and DMPP treatment relative to vehicle treated HFD mice (Fig. 6i, j). These long-term glycemic benefits were further improved when DMPP and icilin were coadministered (Fig. 6i, j). None of the treatments affected circulating lipoprotein fractions (Fig. 6k), but both DMPP and icilin monotherapies improved diet-induced hepatic steatosis (Fig. 6l) and diet-induced NAFLD-induced NASH at thermoneutral conditions (Fig. 6m, n). The combination of DMPP and icilin was superior to the monotherapies with respect to improving NAFLD and NASH. Several genes associated with cholesterol and triglyceride uptake, as well as bile acid metabolism, were altered by the treatments (Supplementary Fig. 7a, b). Notably, Cyp7b1 was highly induced by the combination of DMPP and icilin (Supplementary Fig. 7a). Plasma levels of alanine aminotransferase (ALT) and aspartate aminotransferase (AST) were unchanged (Supplementary Fig. 7c, d). Together, these data underscore that the combination of DMPP and icilin corrects multiple aspects of the metabolic syndrome inflicted by excess consumption of dietary fat and sugar.

### Glycemic benefits are mediated by MC4R

The leptin–melanocortin system plays a key role in the central regulation of glucose metabolism^23^. To test if DMPP signals via the central leptin–melanocortin system to improve glycemia, we used MC4R KO mice. In the DIO MC4R KO mice, neither the monotherapies nor the combination of DMPP and icilin ameliorated glucose intolerance (Fig. 7c, d). UCP1 is required for sympathetic-induced BAT glucose utilization^24^. Here, we found that combination of DMPP and icilin improved diet-induced glucose intolerance equally in DIO WT and DIO UCP1 KO mice (Fig. 7e–h). The benefits of DMPP and icilin cotreatment on glucose tolerance were also preserved in betaless mice (Supplementary Fig. 8a, b) and in TRPM8 KO mice (Supplementary Fig. 8d, e). Together, these data underline that both the glycemic and weight-lowering benefits linked to dual TRPM8 and nAChR α3β4 agonism are centrally mediated and require key signaling nodes in the leptin–melanocortin pathway.

## Discussion

Inspired by the efficiency of two canonical environmental modulators of human energy metabolism—tobacco smoking and cold exposure—to suppress appetite and increase energy expenditure, respectively, we here explored a novel pharmacological strategy in which we aimed to simultaneously mimic the metabolic benefits of both phenomena through a “biochemical cigarette”. We report that the small molecule icilin stimulates the cold receptor TRPM8 to elicit thermogenesis and lower body weight without influencing appetite. In parallel, we discovered that the nAChR α3β4 agonist DMPP suppresses appetite and reverses diet-induced glucose intolerance. Finally, we show that concerted treatment with icilin and DMPP elicits complementary metabolic benefits to reverse obesity, glucose intolerance, and hepatic steatosis in mouse models of obesity and glucose intolerance.

Targeting TRPM8 to increase energy expenditure as a means to combat obesity has previously been suggested^10,19,25–27^. Now, by revealing a potent anti-obesity potential of the TRPM8 agonist icilin, we add substantial support to this idea. In contrast to previous studies that report a cell autonomous effect of TRPM8 on adipocyte thermogenesis, we did not detect TRPM8 expression in adipocytes, nor did we observe any functional effect of icilin on BAT respiration or UCP1 induction. Conversely, our data propose that the ability of TRPM8 activity to increase energy expenditure is indirect—likely implicating sensory neurons and SNS-induced thermogenesis^28^. This is supported by the MSOT data showing a clear physiological phenomenon induced in BAT by the cotreatment, as well as by the data showing the blunted weight-lowering efficacy following cotreatment in the betaless mice. Importantly, the metabolic benefits of DMPP and icilin cotreatment were preserved under thermoneutral conditions. In contrast to previous work on pharmacological β-adrenergic stimulation^22^, we observed that food intake was no longer suppressed following combination therapy at thermoneutrality. Instead, the synergistic weight loss induced by DMPP and icilin cotreatment at thermoneutrality was accompanied by a profound induction of brown fat UCP1 levels. Although it is still debated what rodent housing temperature most accurately mimics the human condition^29–31^, the effectiveness of DMPP and icilin cotreatment to reverse the metabolic syndrome in DIO mice housed at both ambient room temperature and at thermoneutrality strengthens the translational value of this pharmacological approach.

The implication of hypothalamic α3β4 nAChR activation to elicit POMC activity and food intake suppression via the leptin–melanocortin system^13^ encouraged us to investigate selective agonists for this receptor subtype in a potential combination therapy with TRPM8 agonists. DMPP is an agonist at the human α3β4^32^ and is reported to act as a catecholamine and glucagon secretagogue through ganglionic nAChRs^33^. We identified a robust reduction in food intake following DMPP treatment, which was amplified by adjunctive icilin treatment. In agreement with previous findings showing that weight loss following activation of POMC expressed nAChRs is mediated through MC4R signaling in the PVN^13^, we discovered that the ability of DMPP and icilin cotreatment to lower body weight requires both functional α3β4 nAChRs and MC4Rs. Whereas the combination of DMPP and icilin likely converge to drive feeding via MC4R on PVN neurons, the MC4R-dependent increase in energy expenditure possibly implicates MC4Rs located elsewhere^34,35^. Our findings are thus in agreement with a reported divergence of melanocortin signaling in the control of food intake and energy expenditure^36^. The mechanism of how TRPM8 activation potentiates the anorexigenic mechanisms of α3β4 nAChR activation requires further study.

The ability of DMPP to lower HFD-linked hyperphagia and reverse obesity is comparable to that of other nAChR-agonists, including nicotine as shown here. However, the robust and weight-independent improvement in glycemic control following DMPP treatment might be a unique feature relative to other nAChR agonists. In fact, there is evidence that nicotine worsens glycemic control^37^. Recently, it was reported that UCP1 expression is required for β3-adrenergic receptor (AR)-mediated improvements in glucose metabolism^38^. Here, we find that the ability of DMPP and the combination of DMPP and icilin to reverse diet-induced glucose intolerance is independent of BAT UCP1 induction. Notably, while the effects on glucose tolerance appear to be predominantly driven by DMPP, the addition of icilin is required to maximize the benefits on insulin sensitivity. Supporting that mechanisms proximate to brown fat are driving the improvements in glucose metabolism, the potent glycemic benefits governed by DMPP pharmacology were completely lost in MC4R KO mice. However, mouse models of monogenetic obesity and diabetes exhibit a severe background phenotype, so the lack of pharmacological efficacy must be interpreted with caution. Thus, future studies are required to illuminate the molecular interplay between MC4R and α3β4 nAChR signaling in glucose control and for example if MC4Rs on cholinergic sympathetic preganglionic neurons are implicated in the glycemic benefits of α3β4 nAChR agonists^39^.

It is well established that nicotine affects energy metabolism in humans^40^. However, behavioral and cardiovascular off-target effects compromise the utilization of nAChR-based anti-obesity pharmacotherapies. Insights into the tissue-specific and functional differences of distinct nicotinic receptor subtypes might provide important clues for future optimization of nAChR-targeting compounds. As of today, the study of TRP-channel pharmacology in relation to human energy expenditure has mostly focused on TRPV1^41–44^ whereas the effects of potent TRPM8 targeting pharmacology on human energy expenditure are largely unexplored. One study suggested that a combined targeting of TRPM8 and TRPA1 might be required to relevantly increase human energy expenditure^45^. In parallel with assessing the translational value of co-targeting nAChRs and TRPM8, understanding the underlying mechanisms of actions are vital for the clinical progression of this strategy. A recent study found that cold-induced energy expenditure induction involves hepatic *Cyp7b1*-dependent bile acid synthesis, thereby suggesting a novel BAT-liver axis^46^. Notably, the combination of DMPP and icilin potently increases hepatic *Cyp7b1* expression, leaving the exciting opportunity that cold-mimicking effects on hepatic bile acid alterations contribute to the metabolic benefits of the combination therapy.

In summary, we report that stimulation of TRPM8 represents a novel strategy to pharmacologically mimic the benefits of cold exposure on energy metabolism. The ability of the potent TRPM8 agonist icilin to induce a negative energy state is substantially potentiated by coordinated activation of nAChRs by DMPP. The combination of DMPP and icilin engages central satiety circuits and increases BAT thermogenesis, which ultimately improves diet-induced obesity, glucose intolerance, and hepatic steatosis in mice. Conclusively, these data support the compelling potential in coordinated targeting of TRPM8 and α3β4-nAChRs for the treatment of the related epidemics of obesity, type 2 diabetes, and fatty liver disease.

## Methods

### Animals

For pharmacology studies, six- to eight-week-old male C57Bl6j mice were provided ad libitum access to a high-fat, high sucrose diet (D12331; Research Diets, New Brunswick, NJ, USA). The mice had free access to water and were maintained at 23 °C, with constant humidity and on a 12-h light**–**dark cycle. All mice were housed under specific-pathogen-free (SPF) conditions. Mice were maintained under these conditions for a minimum of 11 weeks before treatment initiation. For the thermoneutral study, mice were maintained at constant 30 °C ambient temperature for four to 7 weeks prior to study initiation. CHRNB4 KO mice were generated as described previously^47^. Homozygous mice were used to generate a colony of mice for pharmacological studies. UCP1 KO and MC4R KO were originally provided from Jackson Laboratory (strain names: B6.129-Ucp1tm1Kz/J; B6;129S4-Mc4rtm1Lowl/J, ME, USA). TRPM8 KO mice were generated as described^48^ and provided from Jackson Laboratory (strain name: B6.129P2-*Trpm8*^tm1Jul^/J). For pharmacological studies, homozygous mice were used to generate the colony of homozygous WT and TRPM8 KO mice. Betaless mice were generated as described previously^49^. Homozygous mice were used to generate a colony of mice for pharmacological studies. Mice were randomly assigned to pharmacological treatment groups based on body weight and body fat. The Animal studies were approved and conducted in accordance to the Danish Animal Experimentation Inspectorate and Animal Ethics Committee of the government of Upper Bavaria, Germany.

### In vivo pharmacology and energy metabolism studies

Compounds were administered in a vehicle of 0.01 N NaOH and 1% Tween-80 and injected subcutaneously (s.c.) 0–2 h before the onset of the dark cycle at the indicated doses at a volume of 5 μl per g body weight. Coadministration of compounds was performed by single formulated injections. Researchers were not blinded to the treatment groups. Body composition (fat and lean mass) was analyzed using a magnetic resonance whole-body composition analyzer (Echo-MRI, Houston, TX, USA). Energy expenditure and home-cage locomotor activity were registered with a combined indirect calorimetry system (TSE PhenoMaster, TSE Systems, Bad Homburg, Germany). After 24 h of acclimatization, O_2_ consumption and CO_2_ production were registered every 10 min for a total of 72 h to assess energy expenditure after daily s.c. administration of the indicated treatments. The multidimensional infrared light beam system determines home-cage locomotor activity with beams scanning the bottom and top levels of the cage resulting in activity expressed as beam breaks.

### Glucose metabolism studies

Glucose tolerance, insulin sensitivity, and pyruvate tolerance were analyzed approximately 20 h after the last compound injections. For glucose and insulin tolerance tests, mice were fasted for 6 h and then challenged by an intraperitoneal injection of 1.5 g or 1.75 g glucose per kg body weight or 0.75 U insulin per kg body weight. Pyruvate tolerance was tested in overnight fasted mice at a dose of 1 g pyruvate per kg body weight. Glucose levels in all tolerance tests were measured in the blood sampled from the tail veins before (0 min) and at 15, 30, 60, and 120 min post injection using a handheld glucometer (Abbott, Wiesbaden, Germany). To measure glucose-stimulated insulin secretion, blood was collected from tail veins into EDTA-coated microvette tubes (Sarstedt, Nümbrecht, Germany) at time points 0, 15 and 60 min post glucose injection.

### Blood parameters

Blood was immediately kept on ice, centrifuged at 3000 g and 4 °C, and plasma was stored at −80 °C. Commercially available kits were used to measure plasma levels of insulin (ALPCO Diagnostics, Salem, NH, USA), ALT, and AST (Thermo Fisher Scientific, Erlangen, Germany) according to the manufacturers’ instructions. For fast performance liquid chromatography of cholesterol distribution in different lipoprotein fractions, plasma of the different treatment groups was pooled (*n* = 7) and measured on two Superose 6 columns connected in series^50^.

### Biochemical analysis

Mice were euthanized using CO_2_ after a timed administration of compound and removal of food 2 h prior to the sacrifice. Tissue samples were collected and immediately snap-frozen in liquid nitrogen or kept on dry ice. For gene expression analysis, RNA was isolated using RNeasy Kit (Qiagen, Hilden, Germany) according to the manufacturers’ instructions. Total RNA was reverse transcribed into cDNA using QuantiTect Reverse Transcription Kit (Qiagen, Hilden, Germany), which includes a gDNA elimination step. Gene expression in BAT, iWAT, and liver was profiled with quantitative real-time PCR using either TaqMan single probes or SYBR green (Thermo Fisher Scientific, Erlangen, Germany). The relative expression of the selected genes was normalized to the reference genes peptidylprolyl isomerase B (Ppib) or hypoxanthine-guanine phosphoribosyltransferase (Hprt). Data on gene expression were screened for singular statistically significant outliers using the maximum normed residual (Grubb’s) test. Sequences of primers and TaqMan probes used are listed in alphabetical order in Supplementary tables 1, 2.

### cFOS immunohistochemistry

For cFOS immunohistochemistry, DIO WT mice or DIO CHRNB4 KO mice were treated with compounds for 3 days. 2 h prior to perfusion, food was removed and mice received a final compound treatment. Mice were sacrificed with CO_2_ and transcardially perfused with saline (0.9% NaCl) followed by 4% paraformaldehyde (PFA) in phosphate buffered saline (pH = 7.4). Brains were isolated and post-fixed in 4% PFA at 4 °C before being equilibrated for 48 h with 30% sucrose in Tris-buffered saline (TBS; pH 7.2). After sectioning into 30 μm coronal sections using a cryostat, brain slices were washed in TBS and incubated overnight at 4 °C with a primary antibody anti-cFOS (rabbit polyclonal SC-52, 1:1000, Santa Cruz Biotechnology, Inc, TX, USA) in a solution containing 0.25% gelatin and 0.5% TritonX-100 in TBS. After serial washes in TBS, sections were incubated with Alexa Fluor 568 donkey-anti-rabbit (1:1000, Molecular Probes, Life Technologies GmbH, Darmstadt, Germany) secondary antibody. Sections were serially washed with TBS, mounted on gelatin-pre-coated glass slides, and cover-slipped for image quantification. Quantification of cFOS immunoreactive (cFos+) cells was performed using ImageJ software. Images of single focal planes were captured at 20X magnification by a BZ-9000 fluorescence microscope (Keyence Corporation Itasca, IL, USA). The number of cFos+ nuclei within the PVN was determined according to the Allen mouse brain atlas. All morphometric analyses were performed without previous knowledge of the experimental group.

### Histopathology

After chronic treatment mice were sacrificed with CO_2_. Formalin fixed BAT, iWAT, and liver samples were embedded in paraffin using a vacuum infiltration processor TissueTEK VIP (Sakura, AV Alphen aan den Rijn, Netherlands). 3 µm thick slides were cut using a HMS35 (Zeiss, Jena, Germany) or HM340E (Thermo Fisher Scientific, Erlangen, Germany) rotatory microtome and H & E staining was performed. For H&E staining, rehydration was done in a decreasing ethanol series, rinsing with tapwater, 2 min Mayers acid Hemalum, bluing in tapwater followed by 1 min EosinY (both Sigma-Aldrich, MO, USA). Dehydration was performed in increasing ethanol series, mounting with Pertex® (Medite GmbH, Burgdorf, Germany) and coverslips (Carl Roth Chemicals, Karlsruhe, Germany). The slides were evaluated independently using a brightfield microscope (Axioplan; Zeiss, Jena, Germany). The hepatic steatosis score is defined as the unweighted sum of the three individual scores for steatosis, lobular inflammation, and ballooning degeneration. Steatosis is graded by the presence of fat vacuoles in liver cells according to the percentage of affected tissue (0: < 5%; 1: 5–33%; 2: 33–66%; 3: > 66%). Lobular inflammation is scored by overall assessment of inflammatory foci per 200x field (0: no foci; 1: < 2 foci; 2: 2–4 foci; 3: > 4 foci). The individual score for ballooning degeneration ranges from 0 (none), 1 (few cells) to 2 (many cells). Total scores range from 0 to 8 with scores < 2 considered non-steatosis, 3 considered as borderline steatosis, 4–5 considered onset of steatosis, and > 6 considered steatosis.

For UCP1 immunoreactivity, 3 µm sections of iWAT and BAT samples were stained on a Discovery XT automated stainer (Roche Diagnostics, Mannheim, Germany) employing rabbit anti-UCP1 antibody (1:1500; ab 10983, Abcam, Cambridge, UK). Signal detection was performed using biotinylated goat anti-rabbit (1:750, BA-1000, Vector Laboratories, Burlingame, CA, USA) as a secondary antibody and Dako detection kit (K5001; Agilent, Waldbronn, Germany). The stained tissue sections were scanned with an AxioScan.Z1 digital slide scanner (Zeiss, Jena, Germany) equipped with a 20x magnification objective.

Images were evaluated using the commercially available image analysis software Definiens Developer XD 2 (Definiens AG, Munich, Germany). A specific ruleset was developed in order to detect and quantify UCP1 stained tissue. The calculated parameter was the ratio of UCP1 stained tissue.

### Isolation of primary brown adipocytes

Primary brown adipocytes were isolated from six- to eight-week-old male C57Bl6j mice. The brown adipose tissue was minced and digested at 37 °C for 40 min (1 mg/ml Collagenase II (Thermo Fisher Scientific, Erlangen, Germany), 3 U/ml Dispase II (Sigma-Aldrich, Munich, Germany), 0.01 mM CaCl_2_ in PBS). The cell suspension was filtered using a 100 µm cell strainer, centrifuged and resuspended in growth medium (DMEM/F12 1:1 plus Glutamax (Thermo Fisher Scientific, Erlangen, Germany) containing 1% penicillin/streptomycin (Thermo Fisher Scientific, Erlangen, Germany) and 10% heat-inactivated FBS). After a second filtration step (40 µm cell strainer), pre-adipocytes were cultured to confluency in collagen-coated 12-well plates (VWR International GmbH, Darmstadt, Germany) (37 °C, 5% CO_2_). Differentiation was induced with dexamethasone (5 µM), isobutyl-methylxanthine (0.5 mM), rosiglitazone (1 µM), indomethacin (125 µM), T3 (1 nM), and insulin (0.5 µg/ml) in growth medium. After 2 days of induction, adipocytes were cultured in growth medium containing rosiglitazone, T3, and insulin and at day 4 of differentiation, cells were grown in growth medium with T3 and insulin.

The study involving adipocytes derived from human BAT biopsies was reviewed and approved by the ethics committee of Maastricht University Medical Center (METC 10-3-012, NL31367.068.10, NCT03111719). Informed participant consent was obtained prior to surgery and all ethical regulations were followed. Human BAT cultures were generated similarly to previous work^51^. In short, the stromal vascular fraction was obtained from BAT from an individual undergoing deep neck surgery. Differentiation was initiated for 7 days via differentiation medium made up out of biotin (33 mM), pantothenate (17 mM), insulin (100 nM), dexamethasone (100 nM), IBMX (250 mM), rosiglitazone (5 mM), T3 (2 nM), and transferrin (10 mg/ml). Cells were transferred maintenance medium consisting of biotin (33 mM), pantothenate (17 mM), insulin (100 nM), dexamethasone (10 nM), T3 (2 nM), and transferrin (10 mg/ml) for another 5 days.

### Primary brown adipocytes, gene expression

For gene expression analysis in differentiated primary brown adipocytes, cells were treated with isoproterenol (Sigma-Aldrich, Munich, Germany, 1 µM), icilin (1 and 10 µM) or control (0.1% DMSO) at day 6 of differentiation for 6 h in serum-free growth medium. Medium was removed and the cell plates were snap-frozen at −80 °C until RNA isolation.

### Bioenergetics analysis

Primary brown adipocytes were cultured and differentiated on a XF96^e^-well plate. At day 5 of differentiation, cells were washed and incubated in DMEM XF Assay medium (Seahorse Bioscience, Santa Clara, CA, USA), supplemented with 25 mM glucose (Carl Roth, Karlsruhe, Germany) and 1.5% fatty acid free-BSA (Sigma-Aldrich, Munich, Germany) at 37 °C in a non-CO_2_ incubator for 10 min. Tenfold higher concentrated compounds, dissolved in DMEM XF Assay medium without supplements, were loaded into the ports of a XF Assay Cartridge. Oxygen consumption rate (OCR) was measured using an extracellular flux analyzer (XF96, Seahorse Bioscience, Santa Clara, CA, USA). Basal OCR was recorded for 9 min, followed by measurement of OCR after injection of oligomycin (2 µg/ml, 23 min), norepinephrine (1 µM, 27 min), icilin (1 µM, 27 min) or control (0.1% DMSO, 27 min), carbonyl cyanide-p-trifluoromethoxyphenylhydrazone (FCCP) (1 µM, 14 min), rotenone (2.5 µM)/ antimycin A (2.5 µM)/2-deoxyglucose (10 mM) (9 min). For normalization, the cell plate was fixed with 4% paraformaldehyde and subsequently co-stained with 4′,6-diamidino-2-phenylindole (Dapi) and Nile red. Using a PheraStar plate reader, the fluorescence signal was detected and the bioenergetics measurements were corrected for cell number and differentiation.

### Multi-spectral Optoacoustic Tomography

Multi-spectral Optoacoustic Tomography (MSOT) measurements were conducted with a 256-channel real-time imaging MSOT scanner^52^ (inVision 256-TF, iThera Medical GmbH, Munich, Germany) equipped with a tunable (wavelength range: 680–960 nm) pulsed (pulse duration: < 10 ns) optical parametric oscillator laser with a 10 Hz repetition rate. A fiber bundle was used for delivering homogeneous light along a line of illumination surrounding the animal body. Optoacoustic signals were acquired by a 256-element, cylindrically focused transducer array covering a solid angle of 270° around the imaged animal. The individual detector elements had a central frequency of 5 MHz. The system can acquire cross-sectional (transverse) images of oxygenated and deoxygenated hemoglobin over time. Processing of these images can yield maps of total blood volume, tissue oxygen saturation, and of their transients. A moving stage enables the imaging of different transverse planes, while the illumination and ultrasound detection components remain static.

### Mouse MSOT measurements in vivo

C57Bl6j mice (Charles River Laboratories Inc, Charleston, USA) were anesthetized by i.p. injection of 139 mg/kg ketamine and 6.8 mg/kg xylazine and placed in the MSOT sample holder as described earlier^53^. In brief, each animal was placed onto a thin, polyethylene membrane and positioned in the water bath maintained at 34 °C. Temperature controlled water provided acoustic coupling and maintained animal temperature while imaging. For imaging brown adipose tissue, vehicle, DMPP, icilin or the combination of DMPP and icilin were injected i.p. 40 min before imaging. A total of six animals were used for the activation experiments. For each measurement, multiple images at 10 different wavelengths spanning 700 nm to 900 nm with 20-nm steps were recorded. Preliminary data processing was performed using the commercial suite provided by the manufacturing company (ViewMSOT, Xvue Ltd, Greece). Finally, a model-based image reconstruction method was applied on the raw optoacoustic signals, followed by a spectral unmixing step to calculate the saturation maps over selected regions of interest. Eigenspectra optoacoustic tomography achieves quantitative blood oxygenation imaging deep in tissues^54^.

### Statistics

Statistical analyses were performed on data distributed in a normal pattern using one- or two-way ANOVA followed by Tukey post-hoc analysis as appropriate or an unpaired two-tailed Student’s *t* test. No statistical methods were used to predetermine sample size for in vivo pharmacology studies. Mean energy expenditure was analyzed using ANCOVA with body weight as covariate as previously suggested^55^. All results are presented as mean ± SEM, and *P* < 0.05 was considered significant.

## Electronic supplementary material

Supplementary Information

## Data Availability

The authors declare that all data supporting the findings of this investigation are available within the article, its Supplementary Information, and from the corresponding authors upon reasonable request.
