# Publisher Correction: Coordinated targeting of cold and nicotinic receptors synergistically improves obesity and type 2 diabetes

**DOI:** 10.1038/s41467-018-07479-1

**Published:** 2018-11-20

**Authors:** Christoffer Clemmensen, Sigrid Jall, Maximilian Kleinert, Carmelo Quarta, Tim Gruber, Josefine Reber, Stephan Sachs, Katrin Fischer, Annette Feuchtinger, Angelos Karlas, Stephanie E. Simonds, Gerald Grandl, Daniela Loher, Eva Sanchez-Quant, Susanne Keipert, Martin Jastroch, Susanna M. Hofmann, Emmani B. M. Nascimento, Patrick Schrauwen, Vasilis Ntziachristos, Michael A. Cowley, Brian Finan, Timo D. Müller, Matthias H. Tschöp

**Affiliations:** 1 Institute for Diabetes and Obesity, Helmholtz Diabetes Center (HDC), Helmholtz Zentrum Muenchen & German Center for Diabetes Research (DZD), Neuherberg, Germany; 2 Novo Nordisk Foundation Center for Basic Metabolic Research, Faculty of Health and Medical Sciences, University of Copenhagen, Copenhagen, Denmark; 3 Division of Metabolic Diseases, Department of Medicine, Technische Universität München, Munich, Germany; 4 Section for Molecular Physiology, Department of Nutrition, Exercise and Sports, Faculty of Science, University of Copenhagen, Copenhagen, Denmark; 5 Institute of Biological and Medical Imaging, Helmholtz Zentrum München, German Research Center for Environmental Health (GmbH), Neuherberg, Germany; 6 Chair for Biological Imaging, Technical University of Munich, Munich, Germany; 7 Institute of Diabetes and Regeneration Research, Helmholtz Zentrum Muenchen, German Research Center for Environmental Health (GmbH), Neuherberg, Germany; 8 German Center for Diabetes Research (DZD), Neuherberg, Germany; 9 Medizinische Klinik und Poliklinik IV, Klinikum der Ludwig Maximilian Universität (LMU), Munich, Germany; 10 Research Unit Analytical Pathology, Helmholtz Zentrum München, Neuherberg, Germany; 11 Department of Physiology, and Biomedicine Discovery Institute, Monash University, Clayton, Australia; 12 Department of Human Biology and Human Movement Sciences, NUTRIM School for Nutrition and Translational Research in Metabolism, Maastricht University Medical Center, Maastricht, Netherlands

Correction to: *Nature Communications* 10.1038/s41467-018-06769-y; published online 23 October 2018

In the original PDF version of this article, affiliation 1, ‘Institute for Diabetes and Obesity, Helmholtz Diabetes Center (HDC), Helmholtz Zentrum Muenchen & German Center for Diabetes Research (DZD), Neuherberg, Germany’, was incorrectly given as ‘Institute of Diabetes and Regeneration Research, Helmholtz Zentrum Muenchen, German Research Center for Environmental Health (GmbH), Neuherberg, Germany ‘. This has now been corrected in the PDF version of the article; the HTML version was correct at the time of publication.

