# Supplementary material for: Coordinated targeting of cold and nicotinic receptors synergistically improves obesity and type 2 diabetes
Source: Nat Commun. 2018 Oct 23;9:4304. doi: 10.1038/s41467-018-06769-y (PMC6199300; doi:10.1038/s41467-018-06769-y)
Supplement: Supplementary file 1 — Supplementary Information [file 41467_2018_6769_MOESM1_ESM.pdf]

**SUPPLEMENTAL MATERIAL**

# Coordinated Targeting of Cold and Nicotinic Receptors Synergistically Improves Obesity and Type 2 Diabetes

Christoffer Clemmensen, Sigrid Jall, Maximilian Kleinert, Carmelo Quarta, Tim Gruber, Josefine Reber, Stephan Sachs, Katrin Fischer, Annette Feuchtinger, Angelos Karlas, Stephanie E. Simonds, Gerald Grandl, Daniela Loher, Eva Sanchez-Quant, Susanne Keipert, Martin Jastroch, Susanna M. Hofmann, Emmani B.M. Nascimento, Patrick Schrauwen, Vasilis Ntziachristos, Michael A. Cowley, Brian Finan, Timo D. Müller and Matthias H. Tschöp

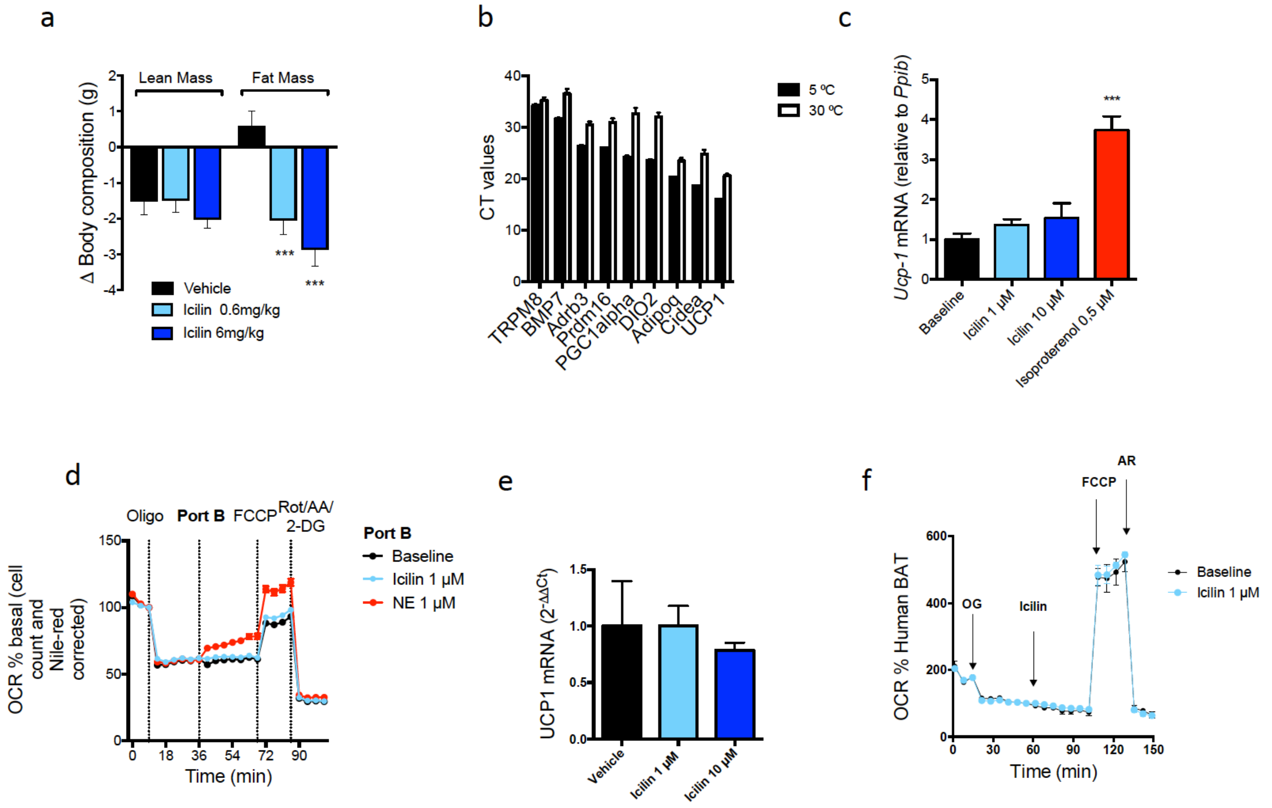


**Supplemental Figure 1. TRPM8 is not expressed in murine brown adipocytes and icilin has no direct effect on brown fat thermogenesis in mouse or human primary adipocytes**

(a) Effect on body lean and fat mass after daily s.c. administration to DIO male C57Bl6j mice of vehicle (black, n=8), icilin 0.6 mg/kg (light blue, n=8), or icilin 6 mg/kg (dark blue, n=6) for 14 days. (b) CT values of gene transcripts in BAT of female warm (30°C, n=8) and cold (5°C, n=7) acclimatized mice. (c) Effects of vehicle (black, n=3), icilin 1 µM (light blue, n=3), icilin 10 µM (dark blue, n=3), or isoproterenol (red, n=3) on UCP1 mRNA levels in primary brown adipocytes from C57Bl6j mice. (d) Effects of vehicle (black, n=22), icilin 1 µM (light blue, n=22), or norepinephrine 1 µM (red, n=24) on oxygen consumption rate (OCR) of primary brown adipocytes from C57Bl6j mice. (e) Effect of vehicle (black, n=3), icilin 1 µM (light blue, n=3), or icilin 10 µM (dark blue, n=3) on UCP1 mRNA expression in cultured adipocytes derived from human BAT. (f) Effects of vehicle (black, n=3) and icilin 1 µM (light blue, n=3) on OCR of primary adipocytes derived from human BAT. ***$p < 0.001$ by one-way ANOVA (a, c, e) with Tukey post-hoc test. All data are presented as mean ± SEM.

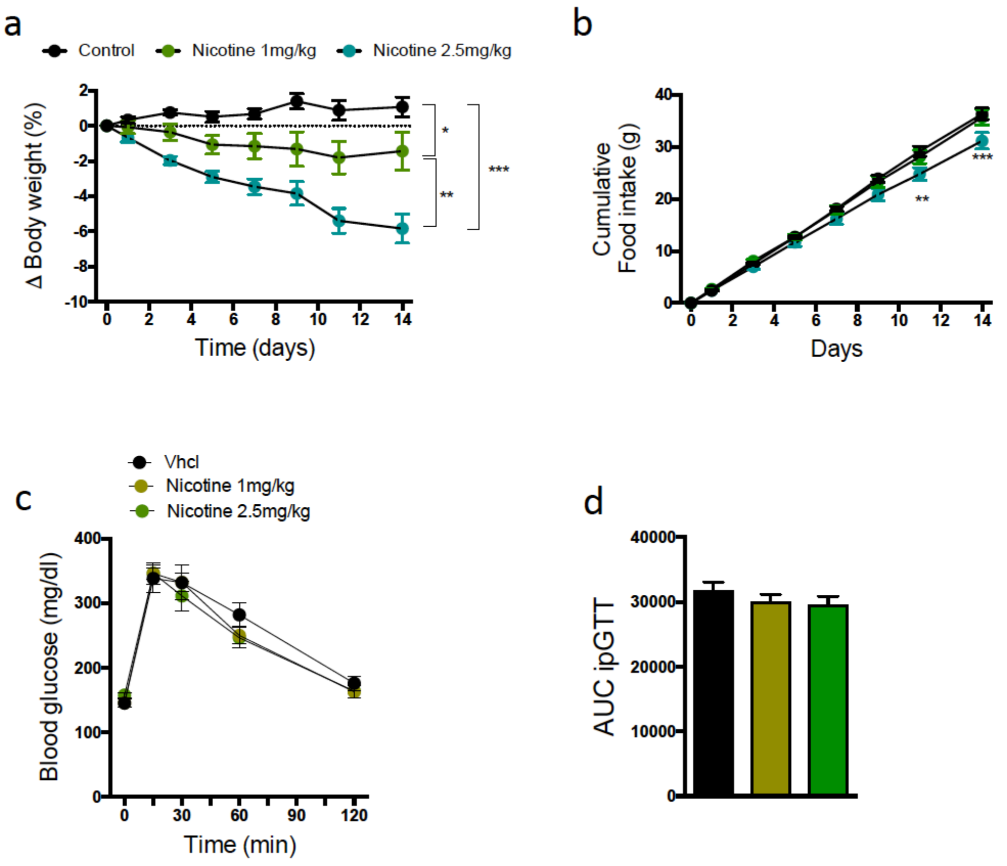


**Supplemental Figure 2. Nicotine lowers body weight but has no impact on glucose tolerance in diet-induced obese mice**

(a) Effects on body weight, (b) food intake, and (c-d) glucose tolerance following daily s.c. injections to DIO male C57Bl6j mice of vehicle (black, n=8), nicotine 1 mg/kg (green, n=8), and nicotine 2.5 mg/kg (turquoise, n=8) for 14 days. *$p < 0.05$, **$p < 0.01$, ***$p < 0.001$ by two-way ANOVA (a, b, c) and one-way ANOVA (d) with Tukey post-hoc test. All data are presented as mean ± SEM.

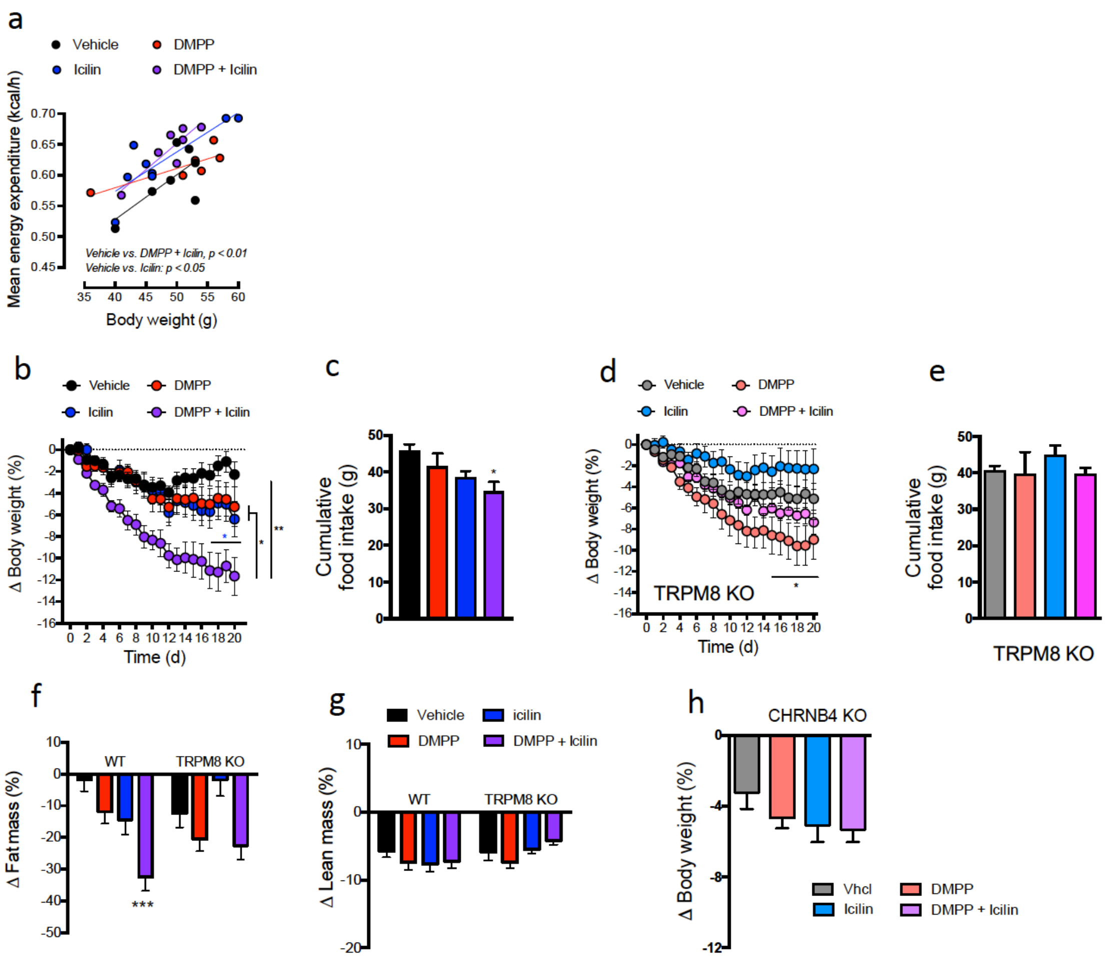


**Supplemental Figure 3. Chronic DMPP and icilin co-administration increases energy expenditure by activating TRPM8 and CHRNB4**

(a) Effect on mean energy expenditure with body weight as covariate following daily s.c. injections to DIO male C57Bl6j mice of vehicle (black, n=7), DMPP 5 mg/kg (red, n=6), icilin 5 mg/kg (blue, n=8), or the combination of DMPP 5 mg/kg and icilin 5 mg/kg (purple, n=7) for 3 days. (b) Effects on body weight and (c) cumulative food intake in DIO male WT mice compared to (d) effects on body weight and (e) cumulative food intake in DIO male TRPM8 KO mice. (f-g) Effects on fat and lean mass following daily s.c. injections to DIO WT and TRPM8 KO mice of vehicle (black, n=8/grey, n=8), DMPP 5 mg/kg (red, n=8/light red, n=8), icilin 5 mg/kg (blue, n=8/light blue, n=8), or the combination of DMPP 5 mg/kg and icilin 5 mg/kg (purple, n=8/pink, n=8) for 21 days. (h) Effects on body weight in DIO male CHRNB4 KO mice treated daily with s.c. injections of vehicle (grey, n=7), DMPP 5 mg/kg (light red, n=6), icilin 5 mg/kg (light blue, n=7), or the combination of DMPP 5 mg/kg and icilin 5 mg/kg (light purple, n=7) for 7 days. *p < 0.05, **p < 0.01, ***p < 0.001 by ANCOVA (vehicle vs. DMPP: p=0.52; vehicle vs. icilin: p=0.048, vehicle vs. DMPP/icilin: p=0.009), two-way ANOVA (b, d), and one-way ANOVA (c, e, f, g, h) with Tukey post-hoc test. All data are presented as mean ± SEM.

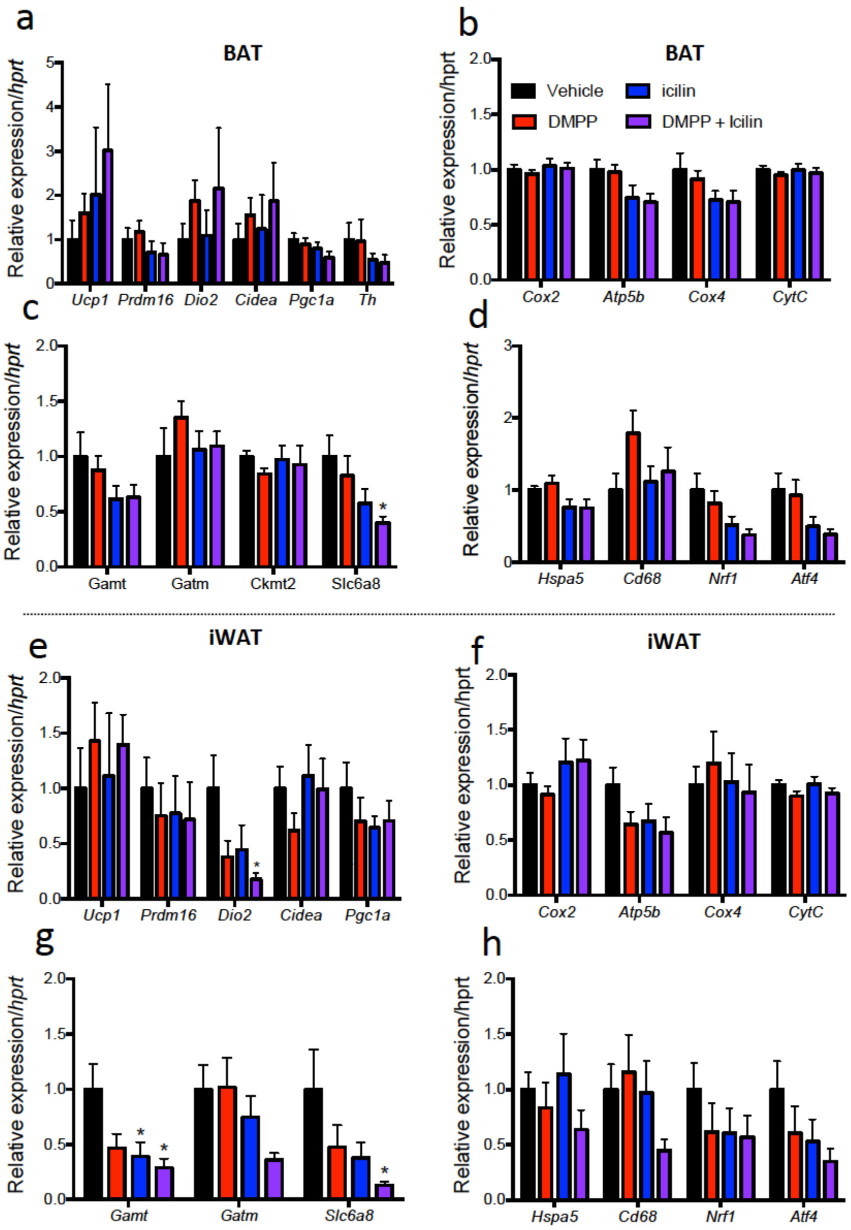


**Supplemental Figure 4. Effects of sub-chronic administration of DMPP and icilin on thermogenic gene programs in DIO WT mice**

Expression of genes in the BAT involved in (a) thermogenesis, (b) mitochondrial respiration chain, (c) creatine signaling pathway, and (d) proteasomal signaling in DIO male C57Bl6j mice. Expression of genes in the iWAT involved in (e) thermogenesis, (f) mitochondrial respiration chain, (g) creatine signaling pathway, and (h) proteasomal signaling in DIO male C57Bl6j mice. Tissues were analyzed following daily s.c. injections of vehicle (black, n=8), DMPP 5 mg/kg (red, n=8), icilin 5 mg/kg (blue, n=8), or the combination of DMPP 5 mg/kg and icilin 5 mg/kg (purple, n=8) for 7 days. *$p < 0.05$ by one-way ANOVA (a-h) with Tukey post-hoc test. All data are presented as mean ± SEM.

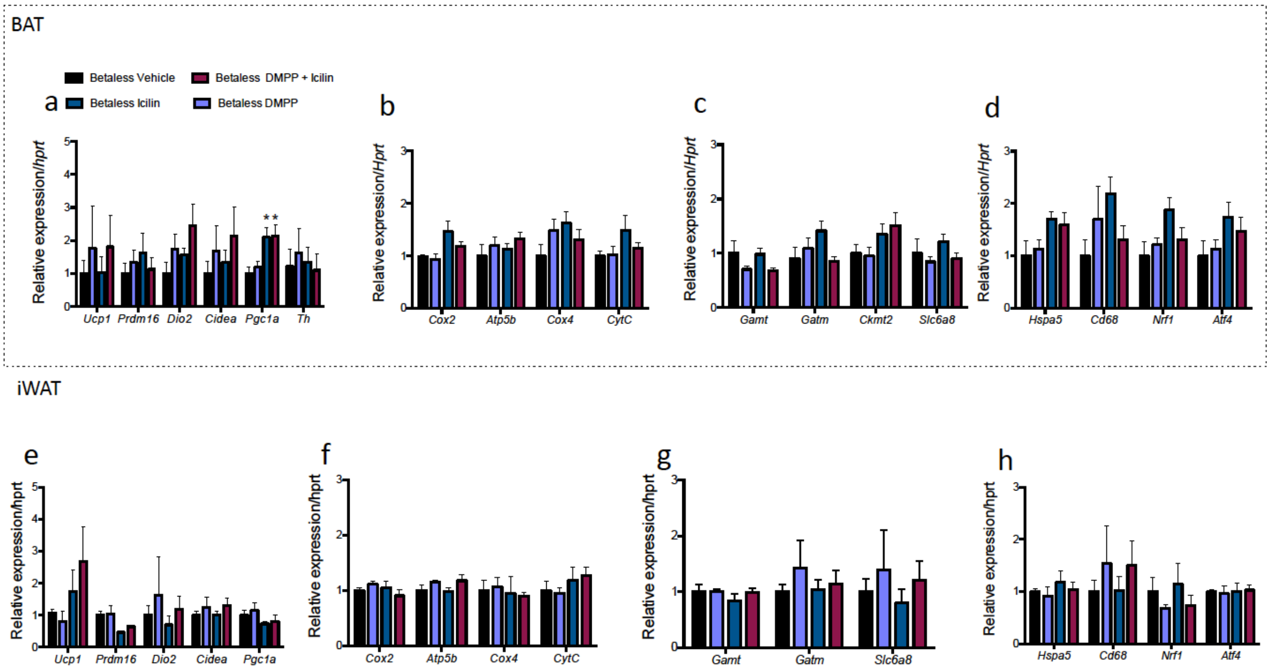


**Supplemental Figure 5. Effects of sub-chronic administration of DMPP and icilin on thermogenic gene programs in betaless mice**

Expression of genes in the BAT involved in (a) thermogenesis, (b) mitochondrial respiration chain, (c) creatine signaling pathway, and (d) proteasomal signaling in DIO male betaless mice. Expression of genes in the iWAT involved in (e) thermogenesis, (f) mitochondrial respiration chain, (g) creatine signaling pathway, and (h) proteasomal signaling in DIO male betaless mice. Tissues were analyzed following daily s.c. injections of vehicle (black, n=6), DMPP 5 mg/kg (orchid, n=6), icilin 5 mg/kg (ocean-blue, n=6), or the combination of DMPP 5 mg/kg and icilin 5 mg/kg (bordeaux, n=6) for 7 days. *$p < 0.05$ by one-way ANOVA with Tukey post-hoc test. All data are presented as mean ± SEM.

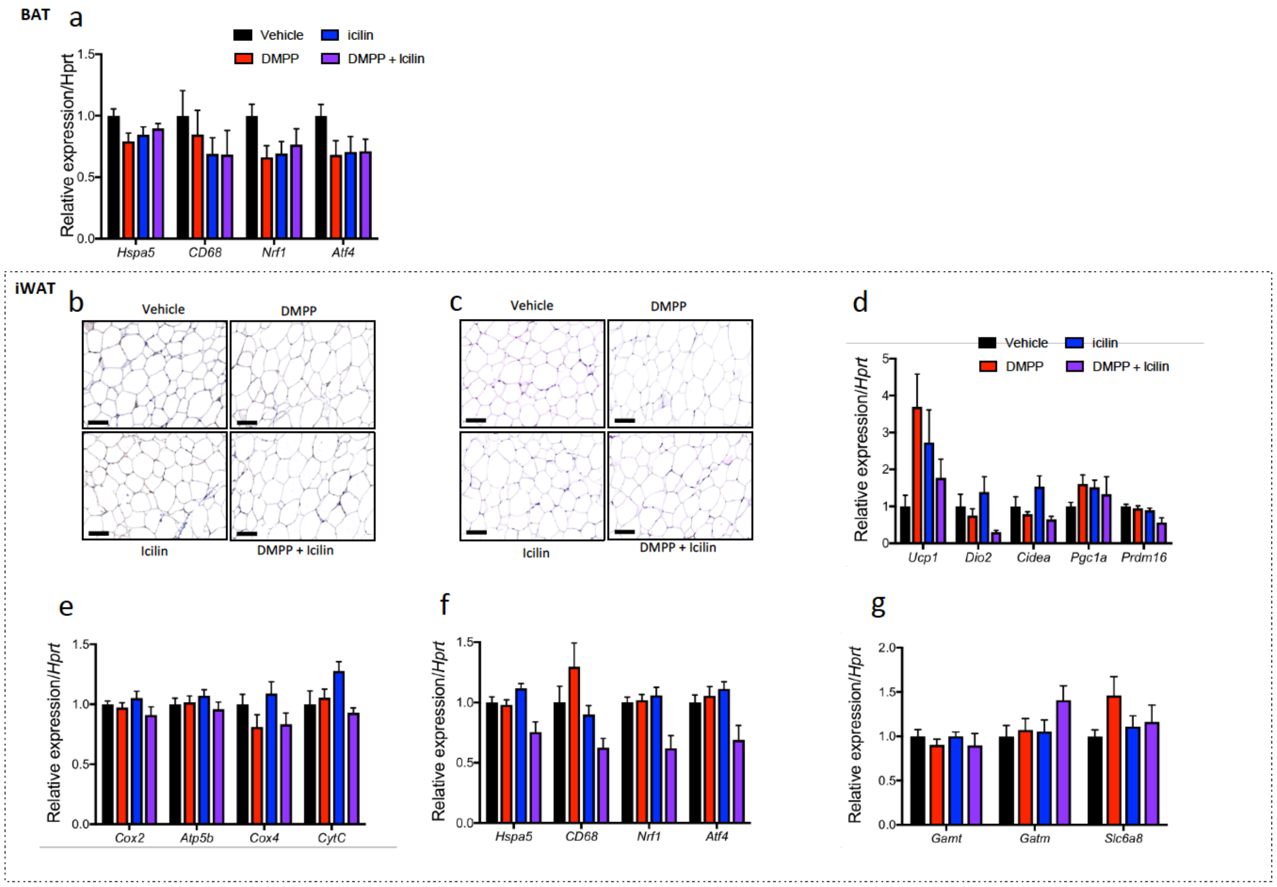


**Supplemental Figure 6. Chronic administration of DMPP and icilin has no impact on iWAT thermogenic programs in DIO WT mice housed at thermoneutral conditions**

(a) BAT expression of genes involved in proteasomal signaling following daily s.c. injections of vehicle (black, n=8), DMPP 5 mg/kg (red, n=7), icilin 5 mg/kg (blue, n=8), or the combination of DMPP 5 mg/kg and icilin 5 mg/kg (purple, n=7) to DIO male C57Bl6j chronically housed at 30ºC for 14 days. (b) UCP1 immunoreactivity staining in iWAT and (c) iWAT H & E staining. iWAT expression of genes involved in (d) thermogenesis, (e) mitochondrial respiration chain, (f) proteasomal signaling, and (g) creatine signaling pathway following daily s.c. injections of vehicle (black, n=8), DMPP 5 mg/kg (red, n=7), icilin 5 mg/kg (blue, n=8), or the combination of DMPP 5 mg/kg and icilin 5 mg/kg (purple, n=7) to DIO male C57Bl6j chronically housed at 30ºC for 14 days. All scale bars are 100µm. Data (a, d, e, f, g) were analyzed by one-way ANOVA with Tukey post-hoc test. All data are presented as mean ± SEM

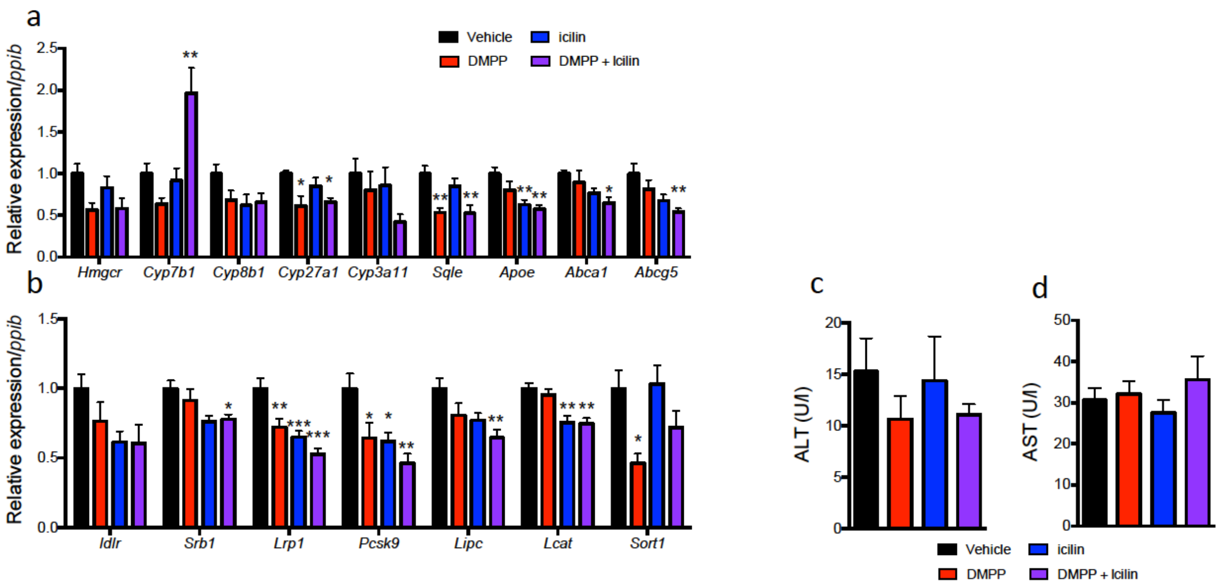


**Supplemental Figure 7. Effects of sub-chronic administration of DMPP and icilin on hepatic gene expression, lipid content, and liver function**

Hepatic expression of genes involved in (a) bile acid metabolism and (b) lipoprotein uptake following daily s.c. injections to DIO male C57Bl6j mice of vehicle (black, n=8), DMPP 5 mg/kg (red, n=8), icilin 5 mg/kg (blue, n=8), or the combination of DMPP 5 mg/kg and icilin 5 mg/kg (purple, n=8) for 7 days. Effects on plasma (c) ALT and (d) AST following daily s.c. injections to DIO male C57Bl6j mice of vehicle (black, n=7), DMPP 5 mg/kg (red, n=7), icilin 5 mg/kg (blue, n=6), or the combination of DMPP 5 mg/kg and icilin 5 mg/kg (purple, n=7) for 21 days *p $< 0.05$, **p $< 0.01$, ***p $< 0.001$ by one-way ANOVA (a-d) with Tukey post-hoc test. All data are presented as mean ± SEM.

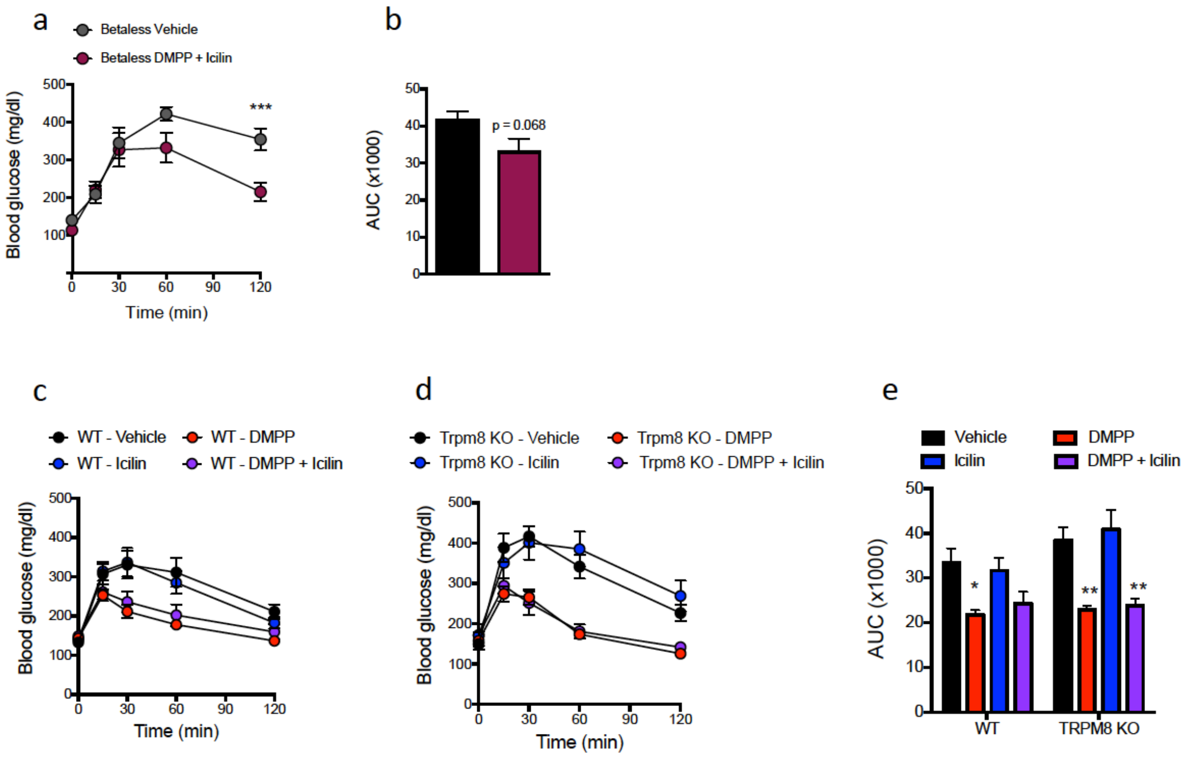


**Supplemental Figure 8. Beta adrenergic receptors and TRPM8 receptors are dispensable for the glycemic benefits of DMPP and icilin co-administration**

(a-b) Effects on glucose tolerance following daily s.c. injections to DIO male betaless mice of vehicle (black, n=6) or the combination of DMPP 5 mg/kg and icilin 5 mg/kg (bordeaux, n=6) for 7 days. (c-e) Effects on glucose tolerance following daily s.c. injections to DIO male TRPM8 WT and KO mice of vehicle (black, n=7 (WT) and n=8 (KO)), DMPP 5 mg/kg (red, n=8), icilin 5 mg/kg (blue, n=8), or the combination of DMPP 5 mg/kg and icilin 5 mg/kg (purple, n=8) for 21 days. *p < 0.05, **p < 0.01, ***p < 0.001 by two-way ANOVA (a, c, d), one-way ANOVA (e) with Tukey post-hoc test, or two-tailed Student's *t*-test (b). All data are presented as mean ± SEM.

## Supplemental Table 1: List of primers

| Gene name | Forward sequence (5' – 3') | Reverse sequence (3' – 5') |
|---|---|---|
| **Abca1** | AAAACCGCAGACATCCTTCAG | CATACCGAAACTCGTTCACCC |
| **Abcg5** | GTACATCGAGAGTGGCCAGA | CTGTGTATCGCAACGTCTCG |
| **Apoe** | GATCAGCTCGAGTGGCAAAG | TAGTGTCCTCCATCAGTGCC |
| **Atf4** | GATGAGCTTCCTGAACAGCG | GCCAAGCCATCATCCATAGC |
| **Atp5b** | CCGGGCAAGAAAGATACAGC | GTCCCACCATGTAGAAGGCT |
| **Cd68** | ACAAAACCAAGGTCCAGGGA | ATTCTGCGCCATGAATGTCC |
| **Cidea** | AATGGACACCGGGTAGTAAGT | CAGCCTGTATAGGTCGAAGGT |
| **Cox2** | GCCCTTCAAGCTCCTAGGTA | CTGGATCCTCTGCTTAGCGA |
| **Cox4** | CTAGAGGGACAGGGACACAC | TGGTTCATCTCTGCGAAGGT |
| **Cyp3a11** | CTCTCACTGGAAACCTGGGT | TCTGTGACAGCAAGGAGAGG |
| **Cyp7b1** | TCTGGGCCTCTCTAGCAAAC | GCACTTCTCGGATGATGCTG |
| **Cyp8b1** | CAGCGGACAAGAGTACCAGA | TGGATCTTCTTGCCCGACTT |
| **Cyp27a1** | CTTCATCGCACAAGGAGAGC | CCAAGGCAAGGTGGTAGAGA |

| | | |
|---|---|---|
| **CytC** | GTTCAGAAGTGTGCCCAGTG | GTCTGCCCTTTCTCCCTTCT |
| **Dio2** | TGCCACCTTCTTGACTTTGC | GGTTCCGGTGCTTCTTAACC |
| **Hmgcr** | AGCTTGCCCGAATTGTATGTG | TGTGTTGTGAACCATGTGACTTC |
| **Hprt** | AAGCTTGCTGGTGAAAAGGA | TTGCGCTCATCTTAGGCTTT |
| **Hspa5** | GACTGCTGAGGCGTATTTGG | AGCATCTTTGGTTGCTTGTCG |
| **Lcat** | GTGCTCCACTTCTTACTGCG | GAACACATGGTCTTCAGGCC |
| **Ldlr** | TCAGACGAACAAGGCTGTCC | CCATCTAGGCAATCTCGGTCTC |
| **Lipc** | ATGTGGGGTTAGTGGACTGG | TTGTTCTTCCCGTCCATGGA |
| **Lrp1** | AACCTTATGAATCCACGCGC | TTCTTGGGGCCATCATCAGT |
| **Nrf1** | ACCCAAACTGAACACATGGC | GCAGTTACCTCATCAGCTGC |
| **Pgc1a** | AGCCGTGACCACTGACAACGAG | GCTGCATGGTTCTGAGTGCTAAG |
| **Ppib** | GCATCTATGGTGAGCGCTTC | CTCCACCTTCCGTACCACAT |
| **Prdm16** | CCGCTGTGATGAGTGTGATG | GGACGATCATGTGTTGCTCC |
| **Sort1** | ATCCCAGGAGACAAATGCCA | AACCTTCCGCCACAGACATA |
| **Sqle** | TGTTGCGGATGGACTCTTCT | GAGAACTGGACTGGGGTTGA |

## Supplemental Table 2: List of TaqMan probes

| Gene name | Assay ID |
|---|---|
| **Ckmt1** | Mm00438221_m1 |
| **Ckmt2** | Mm01285553_m1 |
| **Gamt** | Mm00487473_m1 |
| **Gatm** | Mm00491879_m1 |
| **Hprt** | Mm01545399_m1 |
| **Ppib** | Mm00478295_m1 |
| **Slc6a8** | Mm0050623_m1 |
| **Trpm8** | Mm01299593_m1 |
| **UCP1** | Mm01244861_m1 |
